# Serum selenium levels and the risk of progression of laryngeal cancer

**DOI:** 10.1371/journal.pone.0184873

**Published:** 2018-01-05

**Authors:** Jan Lubiński, Wojciech Marciniak, Magdalena Muszynska, Ewa Jaworowska, Mieczyslaw Sulikowski, Anna Jakubowska, Katarzyna Kaczmarek, Grzegorz Sukiennicki, Michal Falco, Piotr Baszuk, Magdalena Mojsiewicz, Joanne Kotsopoulos, Ping Sun, Steven A. Narod, Jan A. Lubiński

**Affiliations:** 1 Clinic of Otolaryngology, Pomeranian Medical University, Szczecin, Poland; 2 ReadGene, Grzepnica, Poland; 3 Department of Genetics and Pathology, International Hereditary Cancer Center, Pomeranian Medical University, Szczecin, Poland; 4 Regional Oncology Centre Szczecin, Szczecin, Poland; 5 Institute of Econometric and Statistics, University of Szczecin, Szczecin, Poland; 6 Women’s College Research Institute, Toronto, Canada; 7 Dalla Lana School of Public Health, University of Toronto, Toronto, Canada; Beijing Cancer Hospital, CHINA

## Abstract

**Background:**

Observational studies have reported an inverse relationship between selenium status (blood or toenail) and the risk of laryngeal cancer; however, the impact of low serum selenium level on survival has not been evaluated.

**Methods:**

We conducted a prospective study of 296 patients diagnosed with laryngeal cancer in Szczecin, Poland. Serum selenium was measured at diagnosis and prior to treatment. Patients were followed from the date of diagnosis to death at five years. Vital status was obtained by linkage to the Polish National Death Registry.

**Results:**

The five-year survival after diagnosis was 82.0% (95% CI: 68% to 91%) for individuals in the highest quartile of serum selenium (> 66.8 μg/L) and was 28.6% (95% CI 19% to 42%) for individuals in the lowest quartile (<50.0 μg/L). In an age- and sex-adjusted analysis, the hazard ratio (HR) for death from all causes was 7.01 (95% CI 3.81 to 12.9) for patients in the lowest quartile of serum selenium, compared to those in the highest quartile. The corresponding multivariate HR was 3.07 (95% CI 1.59 to 5.94).

**Conclusions:**

This study suggests that a selenium level in excess of 70 μg/L is associated with improved outcome among patients undergoing treatment for laryngeal cancer. Further studies are needed to evaluate if selenium supplementation to achieve this level might improve overall prognosis.

## Introduction

Selenium is an essential trace element which is a cofactor for several antioxidant enzymes, and as a result, there has been much interest in the potential health benefits of adequate selenium status [1]. Epidemiologic evidence is still emerging with respect to the full spectrum of the health benefits associated with (high or adequate) selenium status [2, 3]. Collectively, observational studies have generally reported an inverse relationship between selenium status (circulating or toenail levels) and cancer risk[3,4]. In contrast, findings from randomized controlled trials have failed to demonstrate that selenium supplementation reduces overall cancer risk or cancer-related mortality [5,6]; some studies have even indicated an excess risk of prostate, melanoma and non-melanoma skin cancer, lymphoid cancers as well as of type 2 diabetes, thyroid disorders and nervous system disturbances [2,3,6,7].

With respect to smoking-related cancers, several studies have demonstrated the chemopreventive properties of selenium on the risk of the lung and bladder cancer [1, 4–8,9]. Our group has previously shown that low selenium is associated with an increased risk of laryngeal cancer in Poland, a geographic area with endemically low levels of soil selenium[10]. The role of selenium status on cancer risk for head and neck cancers remains uncertain [11–14]. To our knowledge, there have been no studies specifically evaluating the role of low circulating selenium levels and prognosis specifically following a diagnosis of laryngeal cancer. Thus, the effect on progression from laryngeal cancer remains unknown.

Laryngeal cancer patients have poor survival rates and primary treatment involves surgical resection of the tumour [15]. Adjuvant treatment includes radiotherapy and chemotherapy, but is primarily employed to achieve local control and have little impact on survival. It is of interest to identify modifiable factors, including diet and lifestyle, that impact prognosis of this fatal disease. Thus, the goal of the current study was to evaluate whether circulating selenium levels at time of diagnosis was associated with outcome among a cohort of laryngeal cancer patients residing in Szczecin, Poland.

## Materials and methods

### Study population

The current study included 296 patients diagnosed with laryngeal cancer who were treated at one university hospital in Szczecin, Poland within the Pomeranian Medical University between 2009 and 2013. This centre treats more than 90% of patients diagnosed with laryngeal cancer in the West Pomerania region of Poland. Pathologic diagnosis of squamous cell laryngeal cancer was confirmed by review of biopsies at a single central pathology laboratory in Szczecin, Poland. Patients were all treated at the same otolaryngology clinic and were approached to participate in the study at the time of an outpatient visit. The study was approved by the Ethics Committee of the Pomeranian University of Medicine in Szczecin. All eligible patients provided written informed consent for a blood draw specifically for research purposes and for storage of the blood sample in an existing research biobank. Consenting patients were asked to fast for at least four hours prior to blood collection. A blood sample (10 cc) was obtained during the diagnostic workup and was collected into tubes certified for quantification of trace metals (Vacutainer^®^ System, royal blue cap). Blood samples were taken between 8am and 2 pm and were centrifuged within 30 and 120 minutes of collection to separate the serum from the cellular fraction. The serum samples were stored at -80°C until required for the selenium assay.

### Selenium assay

Serum selenium levels were quantified by inductively coupled mass spectroscopy (ICP-MS NexION 350D, Perkin Elmer) using methane for reduction of polyatomic interferences. Calibration standards were prepared by dilution of 10 mg/l Multi-Element Calibration Standard 3 (PerkinElmer Pure Plus, PerkinElmer Life and Analytical Sciences, USA) with reagent blank consisting of 0.65% solution of nitric acid (Merck, Germany) and 0.002% Tryton X-100 (PerkinElmer, USA). Calibration curves were created using four different concentrations: 0.1 μg/l, 0.5 μg/l, 1 μg/l, 2 μg/l. Germanium (PerkinElmer Pure, PerkinElmer Life and Analytical Sciences, USA) was used as an internal standard and ClinChek^®^ Plasma Control Level I (Recipe, Germany) was used as a reference material. Reference material was measured after each of the six samples. If the difference of the reference material measurements was greater than 5%, the entire series was repeated. Each sample was measured in duplicates in different analytical runs. Prior to analysis, all samples were centrifuged (6000 rpm, 15 min) and the supernatant was diluted 100 times with the reagent blank. Technical details, plasma operating settings and mass-spectrometer acquisition parameters are available on request.

### Statistical analysis

We assigned patients to one of four categories of serum selenium levels (quartiles—each quartile consisted of 74 patients) based on the distribution of selenium levels in the entire patient sample. The ranges for the quartiles were lower than previously reported for the general non-cancer population of the region (≤70 μg/L, < 70–80 μg/L, ≥80–90 μg/L, >90 μg/L). This is as expected because low selenium is a risk factor for laryngeal cancer in Poland [7]. We selected the highest quartile as the reference level. We followed the study population from date of diagnosis until date of death or June 9^th^, 2016. Death was established by linkage to the Polish Vital statistics registry. Subjects in the study were linked to the records of the vital statistics Poland using a using a unique eleven digit identification number (PESEL). Death was all-cause mortality because the specific cause of death was not available. The actuarial survival rates were calculated using the Kaplan-Meier method. We modeled the relationship between serum selenium levels on overall survival using a Cox proportional hazards model. The analysis was adjusted for age (continuous), sex, stage (0, I, II, III, IV), radiotherapy (yes/no); chemotherapy (yes/no) and surgery (yes/no). All analyses were conducted using SAS version 9.4 software (SAS Institute, Cary, NC). All *P* values were 2-sided and were considered statistically significant if *P* ≤0.05.

## Results

The characteristics of the 296 laryngeal cancer patients are presented in Table 1. The mean age of diagnosis was 61 years (range 41 to 81 years). The majority of patients were male (86%), 96% had a history of smoking and 41% were diagnosed at stage IV. The majority had surgery (81%), 55% received radiotherapy and 12% received chemotherapy. The overall five-year actuarial survival rate was 56.7% (95% CI 51% to 63%) for the entire cohort.

**Table 1:** Characteristics of the study population (n = 296).

	N	%
**Sex**		
** Male**	253	86
** Female**	42	15
**Age, mean (range)**	60.7(41–81)	
**Smoking status**		
** Yes**	286	97
** No**	10	3.4
**Stage**		
** Stage I**	63	21
** Stage II**	41	14
** Stage III**	70	24
** Stage IV**	122	41
**Radiotherapy**		
** Yes**	162	55
** No**	134	45
**Chemotherapy**		
** Yes**	35	12
** No**	261	88
**Surgery**		
** Yes**	239	81
** No**	57	19

In the entire cohort, the mean serum selenium level was 58.6 μg/L (range 30.3 μg/L to 103.1 μg/L) and the median level was 58.0 μg/L. The mean selenium levels, according to age, sex, stage and various treatments are presented in Table 2. Crude five-year survival rates by serum selenium quartile were 28.6% (quartile 1), 55.0% (quartile 2), 63.4% (quartile 3) and 82.0% (quartile 4). Table 3 summarizes the age- and sex- adjusted hazard ratios (HR) and associated 95% confidence intervals as well as the multivariate HRs, for various factors on overall survival. In the age- and sex-adjusted analysis, stage, radiotherapy, chemotherapy, surgery and selenium were all significant predictors of survival (*P* ≤ 0.002); however, only the relationship between tumour stage (*P*<0.0001), surgery (*P* < 0.0001), and selenium levels (*P*–trend = <0.0001) remained significant in the multivariate model. Compared to quartile 4 (reference), the HRs adjusted for age- and sex- were 7.01 (95% CI: 3.81–12.9) for quartile 1 vs. 4, 2.95 (95% CI: 1.55–5.62) for quartile 2 vs. 4 and 2.15 (95% CI: 1.10–4.20) for quartile 3 vs. 4. The corresponding multivariate HRs were 3.07 (1.59–5.94) for quartile 1, 2.01 (95%CI 1.04–3.87) for quartile 2 and 1.51 (95%CI 0.76–2.98) for quartile, 3 relative to quartile 4.

**Table 2:** Mean selenium levels by subgroup.

Subgroup	N	Mean selenium level (range) μg/L
**Males**	253	58.7 (30.3–103.1)
**Females**	43	58.6 (37.9–77.5)
**Age**		
** 40–50**	25	51.6 (40.9–58.3)
** 51–60**	129	59.1 (31.4–103.1)
** 61–70**	109	59.3 (32.8–88.1)
** 71–81**	33	55.9 (30.3–88.1)
**Smoking**^1^		
** Yes**	286	58.6 (30.3–103.1)
** No**	10	59.4 (37.2–77.4)
**Stage**		
** Stage I**	63	63.8 (38.8–89.7)
** Stage II**	41	63.8 (38.2–100.6)
** Stage III**	70	57.9 (30.3–103.1)
** Stage IV**	122	54.7 (31.4–92.2)
**Radiotherapy**		
** Yes**	162	54.7 (30.3–89.7)
** No**	134	63.5 (35.8–103.1)
**Chemotherapy**		
** Yes**	35	54.3 (36.8–78.0)
** No**	261	59.2 (30.3–103.1)
**Surgery**		
** Yes**	239	60.5 (30.3–103.1)
** No**	57	51.1 (31.4–89.7)

^1^Smoking includes current and past smokers.

**Table 3:** Hazard ratios and 95% confidence intervals for various factors on survival from laryngeal cancer.

	Age- and sex-adjusted			Fully adjusted model		
Risk factor	Hazard ratio	95% CI	*P* -value	Hazard ratio	95% CI	*P* -value
**Age**						
** <60**	1			1		
** 60+**	1.27	0.89–1.81	0.2	1.18	0.81–1.72	0.4
**Sex**						
** Male**	1			1		
** Female**	1.26	0.78–2.05	0.34	1.36	0.83–2.22	0.23
**Stage**						
** Stage I**	1			1		
** Stage II**	1.72	0.71–4.14	0.23	1.77	0.74–4.27	0.2
** Stage III**	2.64	1.27–5.52	0.01	2.3	1.08–4.92	0.03
** Stage IV**	6.76	3.48–13.1	<0.0001	5.25	2.50–11.0	<0.0001
**Radiotherapy**						
** No**	1			1		
** Yes**	4.14	2.71–6.33	<0.0001	1.24	0.72–2.19	0.44
**Chemotherapy**						
** No**	1			1		
** Yes**	2.18	1.34–3.54	0.002	0.8	0.48–1.33	0.39
**Surgery**						
** No**	1			1		
** Yes**	0.25	0.17–0.37	<0.0001	0.32	0.20–0.52	<0.0001
**Selenium**						
**Quartile 4 (66.8–103.1)**	1			1		
**Quartile 3 (58.0–66.2)**	2.15	1.10–4.20	0.03	1.51	0.76–2.98	0.24
**Quartile 2 (50.1–57.9)**	2.95	1.55–5.62	0.001	2.01	1.04–3.87	0.04
**Quartile 3 (30.3–50.0)**	7.01	3.81–12.9	<0.0001	3.07	1.59–5.94	0.0009
***P*—trend**			<0.0001			<0.0001

**Fig 1:**
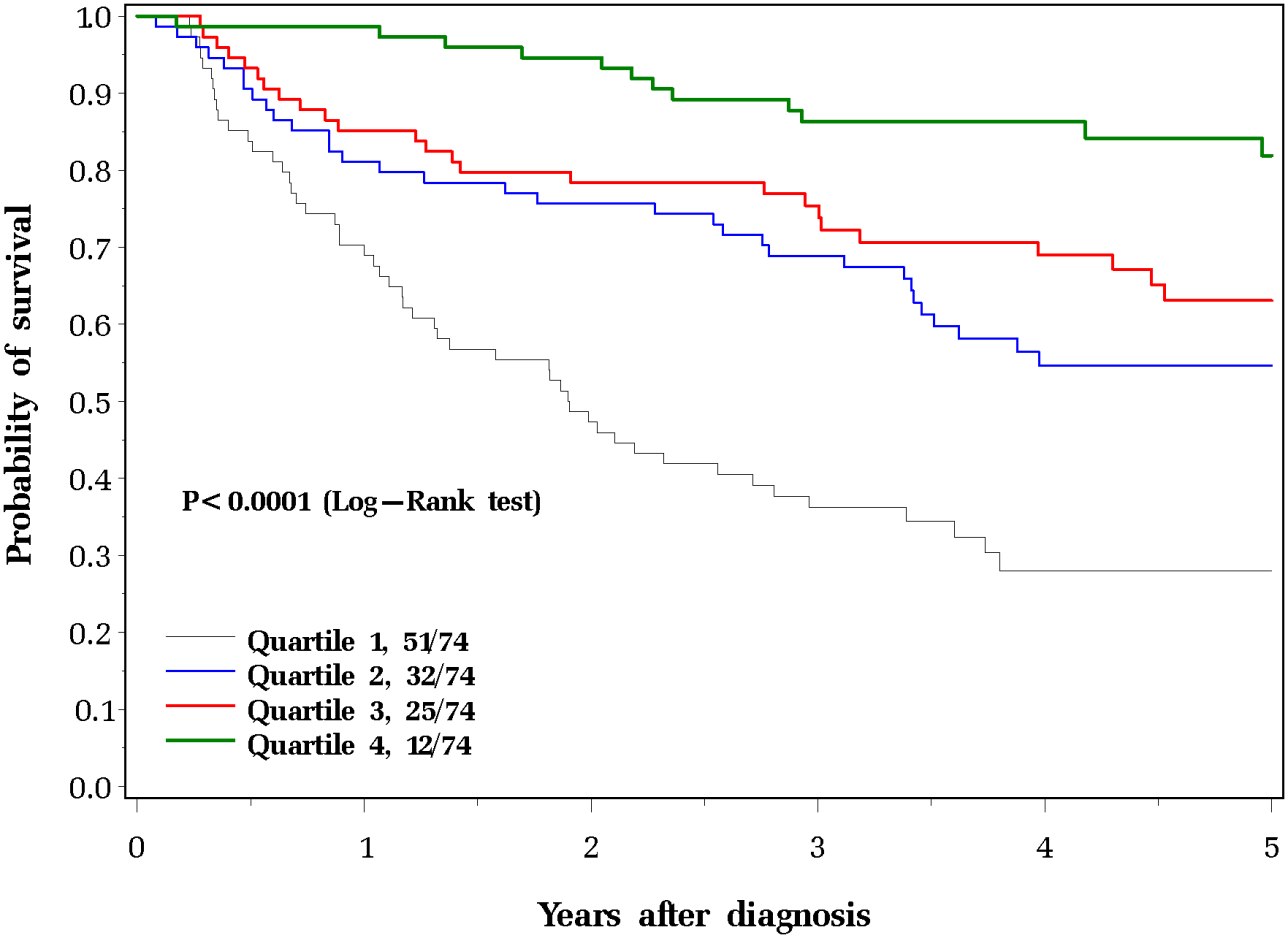
Five-year overall survival among laryngeal patients by serum selenium levels.

The actuarial survival rates, according to quartile of serum selenium, are presented graphically in Fig 1. The effect was present in men (HR = 6.50; 95% CI 3.43 to 12.3) and in women (HR = 13.2; 95% CI 1.61 to 109.2) and in patients with radiotherapy (HR = 6.60; 95% CI 2.61 to 16.7) and in patients without radiotherapy (HR = 2.67; 95% CI 0.87 to 8.25). The hazard ratios for all subgroups are presented in Table 4.

**Table 4:** Age- and sex-adjusted hazard ratios and 95% confidence intervals for low (quartile 1) versus high (quartile 4) serum selenium levels on survival from laryngeal cancer.

Risk factor	Hazard ratio	95% CI	*P*-value
**Sex**			
** Male**	6.5	3.43–12.3	<0.0001
** Female**	13.2	1.61–109.2	0.02
**Stage**			
** Stage I**	16.5	2.42–112.0	0.004
** Stage II**	0.9	0.10–8.17	0.92
** Stage III**	15	1.91–117.3	0.01
** Stage IV**	4.01	1.68–9.58	0.002
**Radiotherapy**			
** Yes**	6.6	2.61–16.7	<0.0001
** No**	2.67	0.87–8.25	0.09
**Chemotherapy**			
** Yes**	5.04	1.02–25.0	0.05
** No**	7.64	3.92–14.9	<0.0001
**Surgery**			
** Yes**	6.56	3.17–13.6	<0.0001
** No**	2.13	0.60–7.53	0.24

## Discussion

The results from this study suggest that low serum selenium levels at the time of diagnosis (prior to treatment) are associated with a significantly increased risk of death among laryngeal cancer patients who reside in Szczecin, Poland. The effect appeared to be continuous; that is, the risk of death declined continuously with increasing circulating levels of selenium and there was no apparent threshold value. Individuals in the lowest quartile of selenium had a three-fold increased risk of death during five years of follow-up. We observed a stronger prognostic role of low selenium status on mortality among those with advanced stage disease (stage III/IV). Together with our earlier observations [10], these findings collectively suggest that low selenium levels are both a risk factor for laryngeal cancer and a prognostic factor in Poland. Selenium supplementation may benefit individuals living in geographical areas with low selenium levels.

Until now, there has been very limited evidence for a role of selenium status on laryngeal cancer progression or outcome. An earlier hospital-based report of 30 head and neck cancer patients reported that patients with serum selenium levels returning to the normal range after one-year of follow-up were more likely to have evident disease compared to those who had persistently lower serum selenium [16]. To our knowledge, there are no other studies that have specifically evaluated the relationship between selenium levels and prognosis from laryngeal cancer; however, based on our findings, further studies are indicated.

In contrast, several studies have examined selenium status and cancer incidence and (overall) cancer mortality. In one study using data from the National Health and Nutritional Examination Survey (NHANES), Goyal *et al*., reported a significant inverse relationship between increasing quintiles of serum selenium levels and all-cause mortality (*P*–trend = 0.03); however, this relationship did not achieve statistical significance in the [17]. Others have reported on the mortality experience of participants in randomized-controlled trials of antioxidant supplements (including selenium), but none specifically reported on head and neck cancers (reviewed in [18]). In a meta-analysis of 68 randomized trials with 23,606 participants, Bjelakovic *et al*., found no clear relationship between selenium supplementation alone or in combination with other antioxidants on overall mortality [18]. In a review of 55 prospective studies and eight randomized controlled trials, Vinceti *et al*., published a significant inverse relationship between high selenium exposure and mortality in the observational studies (summary odds ratio = 0.60; 95%CI 0.39–0.93) but no impact on outcome among randomised trials (summary RR = 0.81; 95%CI 0.49–1.32) [3]. In a meta-analysis of 47 trials of which 8 considered selenium given singly or in combination with others dietary supplements, Schwingshackl *et al*. reported inverse association for selenium and all-cause mortality (RR: 0,93), with no important effect on cancer mortality [19].

Many of the recent trials have shown that selenium does not modify risk of overall and site specific cancer [9, 20–25]. In the most recent review Vinceti *et al*. concludes that hypothesis of an effect of low environmental selenium exposure in increasing cancer risk may now be ruled out [6]. However the fact remains that serum selenium level differs depending on geographic region. Because of variability in soil selenium levels, dietary intake in Europe tends to be lower than in the United States and this is reflected in serum levels at the population level [26,27]. Using data from the National Health and Nutritional Examination Survey (NHANES) conducted in the United States, the mean serum selenium levels for adults 40 years of age or older was 135.9 μg/L for women and 138.6 μg/L for men [28]. Selenium deficiency, characterized as serum levels of < 70 μg/L, is very rare in countries such as the United States and Canada [29]. In the meantime in Poland there are many regions where concentration of selenium in blood plasma reaches levels of 50–55 μg/L[30]. In our study the mean serum selenium level was 58 μg/L, what confirmed our previous findings in the study concerning serum selenium and risk of laryngeal (lung) cancer [10]. Thus, the results from the current analysis are likely not generalizeable to patients residing in countries with ubiquitously high selenium levels.

Selenium is an essential trace element and 25 selenium containing proteins have been identified in humans [31]. Approximately half of the human selenoproteins encode oxido-reductases and thus are critical for cellular redox homeostasis, while the function of the remaining selenoproteins is unknown [31]. The cancer preventive effects, as well as other health benefits, of this micronutrient have primarily been linked to the oxidoreductase function of these selenoproteins, particularly thioredoxin reductase 1 (TR1), 15 kDa selenoprotein (Sep15) and glutathione peroxidase 2 (GPx2) [32]. The daily recommended intake of selenium is based on the amount needed to maximize synthesis of the selenoprotein glutathione peroxidase [29]. It is not clear which physiological function of selenium is related to laryngeal cancer however it is highly possible that selenium can modify cancer progression. Jönsson-Videsäter *et al*. reported the effect of sodium selenite on the induction of apoptosis in lung cancer cell lines [33]. Studies presented by Heras *et al*. have shown that selenium plays a significant role in preventing high grade tumours [34]. Zhang *et al*. reported inverse correlation between serum selenium levels and advanced clinical stages of cervical cancer before treatment [35]. In contrast the studies presented by Kenfield *et al*. have showed that selenium supplementation at dose of 140 μg/day or more may worsen the prognosis in cases of metastatic prostate cancer [36].

There currently are no specific therapeutic agents that are able to modify the survival experience of laryngeal cancer patient to the extent that we observe in Fig 1. In our study, we observed hazard ratios below unity for use of chemotherapy and surgery, but this was not a randomized study and the observed effects may be attributed to patient selection for treatment. The association with selenium level and survival was strong and statistically significant for with stage IV disease. This suggests that there may be a possibility of delaying time to death using selenium in individuals with advanced laryngeal cancers. However, it is too early to tell from these observations that the association between serum selenium and survival is causal or if the effect of low selenium can be mitigated by selenium supplementation and future studies are required to determine if selenium has therapeutic benefit in this setting.

The strengths of our study include the large number of patients identified from one institution and the use of fasting blood samples for selenium measurement. We controlled for predictors of selenium levels as well as risk of laryngeal cancer such as age and smoking status. Despite this, our study was not without limitations. Selenium level was taken after diagnosis (but before treatment) and it is possible that the cancer might have influenced the level. Selenium was measured only once and serum levels tends to fluctuate. Because of limitations in the data in the Polish vital statistics data base we used all-cause mortality as an endpoint. We expect the majority of the deaths (but not all) in this study are due to laryngeal cancer. By using all-cause mortality, we may have underestimated the effect on laryngeal cancer specific mortality. The individual subgroups were small and it is difficult to compare the effects of selenium in various subgroups.

In summary, the results of the current study suggest that the optimum level of selenium that corresponds with improved outcome from laryngeal cancer is above 70 μg/L. Through selenium supplementation, we can expect to achieve a selenium level within in this range. Future studies are needed to evaluate the potential impact of supplementation to this level on mortality from laryngeal cancer.
