# Correction: Serum selenium levels and the risk of progression of laryngeal cancer

**DOI:** 10.1371/journal.pone.0194469

**Published:** 2018-03-12

**Authors:** Jakub Lubiński, Wojciech Marciniak, Magdalena Muszynska, Ewa Jaworowska, Mieczyslaw Sulikowski, Anna Jakubowska, Katarzyna Kaczmarek, Grzegorz Sukiennicki, Michal Falco, Piotr Baszuk, Magdalena Mojsiewicz, Joanne Kotsopoulos, Ping Sun, Steven A. Narod, Jan A. Lubiński

The first author’s name is incorrect. The correct name is: Jakub Lubiński.
